# Delphinidin induces a fast-to-slow muscle fiber type shift through the AMPK signaling pathway in C2C12 myotubes

**DOI:** 10.1016/j.bbrep.2024.101884

**Published:** 2024-11-23

**Authors:** Motoki Murata, Rina Takahashi, Yuki Marugame, Yoshinori Fujimura, Hirofumi Tachibana

**Affiliations:** a Advanced Research Support Center (ADRES), Ehime University, Matsuyama, Ehime, Japan; b Graduate School of Agriculture, Ehime University, Matsuyama, Ehime, Japan; c Division of Applied Biological Chemistry, Department of Bioscience and Biotechnology, Faculty of Agriculture, Kyushu University, Fukuoka, Fukuoka, Japan

**Keywords:** Anthocyanidin, C2C12 myotube, MyHC, AMPK

## Abstract

Delphinidin, a plant anthocyanidin, suppresses disuse muscle atrophy in mice. However, its effect on muscle fiber type shift is unclear. To examine whether delphinidin affects skeletal muscle fiber type, differentiated C2C12 cells were treated with delphinidin. Results revealed that delphinidin upregulated the mRNA expression of myosin heavy chain type I (MyHCI), troponin C1, troponin I1, and MyHCIIx and increased slow MyHC protein level in C2C12 myotubes. Delphinidin also enhanced succinic dehydrogenase (SDH) activities and suppressed lactate dehydrogenase (LDH) activity. Adenosine monophosphate–activated protein kinase (AMPK) inhibition attenuated delphinidin-induced MyHCI upregulation and MyHCIIb downregulation. We investigated the effect of delphinidin on the upstream factors involved in AMPK activation. Delphinidin increased liver kinase B1 (LKB1) phosphorylation and nuclear respiratory factor 1 (NRF1) and calcium/calmodulin-dependent protein kinase 2 (CaMKK2) protein levels. In conclusion, delphinidin induced muscle fiber type conversion from fast-twitch to slow-twitch muscles through the AMPK signaling pathway.

## Highlights

• Delphinidin induces fast-to-slow muscle fiber type shift in C2C12 myotubes.
• Delphinidin activates the AMPK signaling pathway.
• AMPK inhibitor attenuates delphinidin's effect on muscle fiber type shifting.

## Graphical abstract

**Figure:**
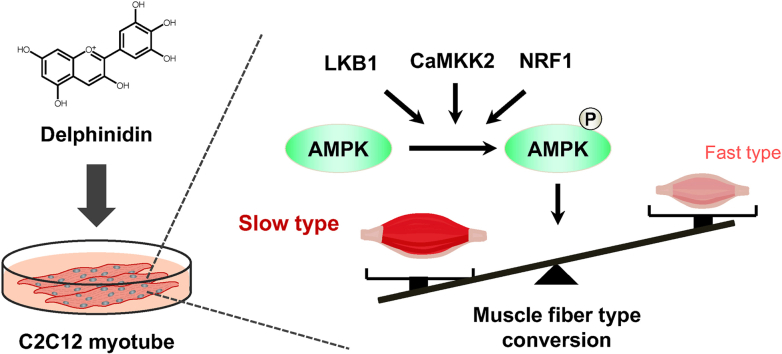

## 1 Introduction

Anthocyanins are plant origin pigments and are naturally occurring polyphenols that found in vegetables, fruits, and flowers [1]. Anthocyanins are glycosylated forms, whereas anthocyanidins are known as the aglycones. Anthocyanidins contribute to human health [2]. Delphinidin, an anthocyanidin, reportedly possesses anticancer [3], anti-inflammatory [4], anti-obesity [5], and cardiovascular protection [6] effects. In our previous report, delphinidin prevented disuse muscle atrophy in mice by increasing the expression of miR-23a, a microRNA that suppresses muscle atrophy [7]. However, the effects of delphinidin on skeletal muscle properties are still unknown.

Skeletal muscle is one of the body's vital organs, and muscle fibers are the basis of muscle composition. Based on differences in myosin heavy chain (MyHC) gene expression, skeletal muscle fibers are divided into slow-twitch (type I) and fast-twitch (type II) [8]. Type II fibers are further classified into three major subtypes: types IIa, IIx, and IIb [9]. The characteristics of types IIa (fast contraction, oxidative) and IIx (fast contraction, glycolytic) are intermediate between those of types I (slow contraction, oxidative) and IIb (fast contraction, glycolytic). Differences in these muscle fiber types exhibit different metabolic and contractile properties. Oxidative metabolism energizes slow-twitch muscles, which show high endurance. Fast-twitch muscle mainly relies on the glycolytic pathway and provides greater powerful forces. Troponin isoforms (troponin C, troponin I) were also found to be markers of muscle fiber types [10]. Moreover, succinate dehydrogenase (SDH), a marker for oxidative capacity, and lactate dehydrogenase (LDH), the glycolytic metabolic enzyme, activities are indicators that characterize muscle fiber types [11,12]. Muscle fiber types can be converted from a slow-twitch to a fast-twitch type and vice versa. It has been reported that muscle fiber types can be regulated by exercise [13], hormone [14] and nutrition [15,16].

Changes in skeletal muscle fiber types are associated with various signaling factors, including adenosine monophosphate–activated protein kinase (AMPK) [17], Sirtuin 1 (Sirt1) [18], and peroxisome proliferator–activated receptor-γ coactivator-1α (PGC-1α) [19]. Some polyphenols, such as quercetin [20] and resveratrol [21], reportedly have effects on skeletal muscle fiber types through the AMPK pathway. In this study, we aimed to examine the effect of delphinidin on skeletal muscle fiber type shift in C2C12 myotubes and its mechanism.

## 2 Materials and methods

### 2.1 Reagents

Pelargonidin (≥97 % pure), cyanidin (≥96 % pure), and delphinidin (≥97 % pure) were purchased from Extrasynthese (Genay, France) and were prepared as stocks in dimethyl sulfoxide (DMSO). Dorsomorphin (compound C) and anti-CaMKK2 antibody were purchased from Abcam (Cambridge, UK). Anti-fast MyHC and anti-slow MyHC antibodies were obtained from Santa Cruz Biotechnology (Heidelberg, Germany). We obtained anti-AMPK, anti-phospho-AMPK (*p*-AMPK), anti-LKB1, anti-phospho-LKB1 (*p*-LKB1), and anti-NRF1 antibodies from Cell Signaling Technology (Berkeley, MA, USA), and anti-β-actin antibody was obtained from Sigma-Aldrich (St. Louis, MO, USA).

### 2.2 Cell culture

C2C12 myoblast cells, which were purchased from the American Type Culture Collection (Manassas, VA, USA), were maintained in Dulbecco's modified Eagle's medium (DMEM, FUJIFILM Wako Pure Chemical, Osaka, Japan) containing 10 % fetal bovine serum (FBS, Gibco, Carlsbad, CA, USA) at 37 °C and 5 % CO_2_. The C2C12 myoblasts were differentiated into myotubes by incubating them in DMEM containing 0.5 % FBS when the cells reached approximately 80 % confluence. After 4 days of differentiation, these C2C12 myotubes were treated with delphinidin.

### 2.3 Quantitative reverse transcription-PCR (qRT-PCR)

Total RNA was extracted from C2C12 myotubes using Tri reagent (Cosmo Bio Co., Tokyo, Japan) following the manufacturer's instructions. For mRNA detection, we used the PrimeScript RT reagent Kit (Takara Bio Inc., Shiga, Japan) to reverse-transcribe total RNA to synthesize cDNA. We conducted qRT-PCR using SsoAdvanced™ Universal SYBR Green Supermix (Bio-Rad Japan Laboratories, Tokyo, Japan) on QuantStudio 3 Real-Time PCR system (Applied Biosystems, Foster City, CA, USA). Our supplementary table (Table S1) shows the specific primer sequences for each gene. The mRNA expression was normalized to β-actin expression.

### 2.4 Western blot

Total protein was isolated from C2C12 myotubes using cell lysis buffer containing 150 mM NaCl, 50 mM Tris-HCl (pH 7.5), 50 mM NaF, 30 mM Na_4_P_2_O_7_, 1 mM EDTA, 1 mM pervanadate, 1 mM phenylmethylsulfonyl fluoride, 2 μg/mL aprotinin, and 1 % Triton X-100. Protein samples were electrophoresed on SDS-polyacrylamide gels and then electroblotted onto a nitrocellulose membrane (GE Healthcare, Chicago, IL, USA). After blocking, we identified proteins by using indicated antibodies in tris-buffered saline with Tween 20 (TTBS) containing 1 % bovine serum albumin. Then, we washed the membranes with TTBS, followed by incubation with anti-mouse or anti-rabbit horseradish peroxidase (HRP) conjugates. Specific bands were detected using TMA-6 (Lumigen, Mile Road, MG, USA) and ChemiDocMP Imaging System (Bio-Rad) according to the manufacturer's instructions. The protein level was quantified using ImageJ software and normalized to β-actin expression.

### 2.5 Metabolic enzyme activities

SDH and LDH activities were assessed using assay kits (BioVision Inc., Milpitas, CA, USA) according to the manufacturer's instructions.

### 2.6 Mitochondrial DNA (mtDNA) quantification

We used mtDNA Extractor® CT Kit (FUJIFILM Wako Pure Chemical) for extracting mtDNA from C2C12 myotubes and conducted qRT-PCR for evaluating the mtDNA content. The mtDNA content was measured using the primers 5′-GCCCATTAAACTTGGGGGTA-3′ and 5′-TTATGTTGGTCATGGGCTGA-3′.

### 2.7 Statistical analysis

Results are expressed as the mean ± standard error of the mean (SEM). Data were analyzed with GraphPad Prism using one-way analysis of variance for more than two groups or Student's t-test for two-group comparisons. A *P*-value less than 0.05 was considered indicating significant.

## 3 Results

### 3.1 Delphinidin induces fast-to-slow muscle fiber type shift in C2C12 myotubes

**Fig. 1:**
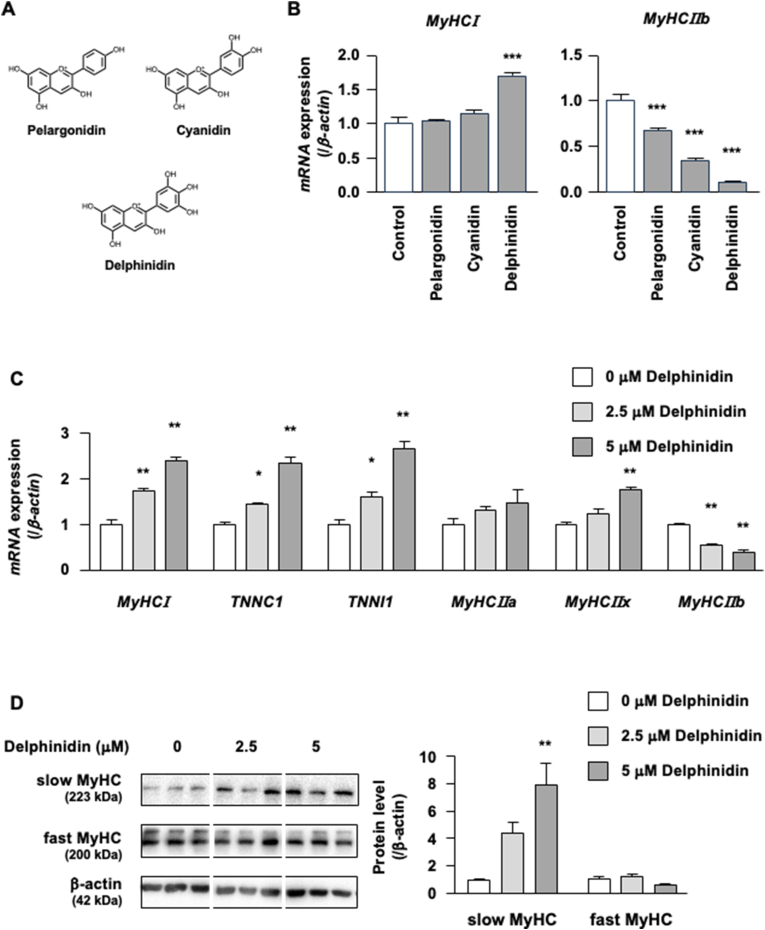
Delphinidin induces muscle fiber type shift in C2C12 myotubes. (A) Chemical structure of typical anthocyanidins. (B) C2C12 myotubes were treated with 5 μM anthocyanidin for 48 h. We conducted qRT-PCR to measure the mRNA expression of MyHCI and MyHCIIb. (C, D) C2C12 myotubes were treated with 2.5 μM or 5 μM delphinidin. After 48 h of treatment, the mRNA expression levels were measured via qRT-PCR. After 72 h of treatment, the protein levels of slow MyHC and fast MyHC were measured by western blotting. Data are presented as mean ± SEM, *n* = 3. ∗*P* < 0.05 and ∗∗*P* < 0.01 as compared with Control or 0 μM delphinidin.

The effect of delphinidin on skeletal muscle fiber type conversion was examined using C2C12 myotubes as an *in vitro* model. We first compared the effects of delphinidin and other anthocyanidins (pelargonidin and cyanidin) (Fig. 1A) on MyHCI and MyHCIIb mRNA expression. Delphinidin increased the mRNA expression of MyHCI, whereas pelargonidin and cyanidin did not (Fig. 1B). Pelargonidin, cyanidin, and delphinidin significantly suppressed the mRNA expression of MyHCIIb (Fig. 1B). Troponin C1 (TNNC1) and troponin I1 (TNNI1) were expressed in the slow-twitch muscle fiber, and they can indicate skeletal muscle proportion. In C2C12 myotubes, delphinidin significantly upregulated MyHCI, TNNC1, TNNI1, and MyHCIIx mRNA expression levels but downregulated MyHCIIb mRNA expression (Fig. 1C). Furthermore, delphinidin significantly increased the protein level of slow MyHC and showed no effect on fast MyHC protein (Fig. 1D). Our previous study found that delphinidin did not modify the viability of C2C12 cells at concentrations of 2.5 μM and 5 μM (data not shown).

### 3.2 Effect of delphinidin on C2C12 myotube characteristics

**Fig. 2:**
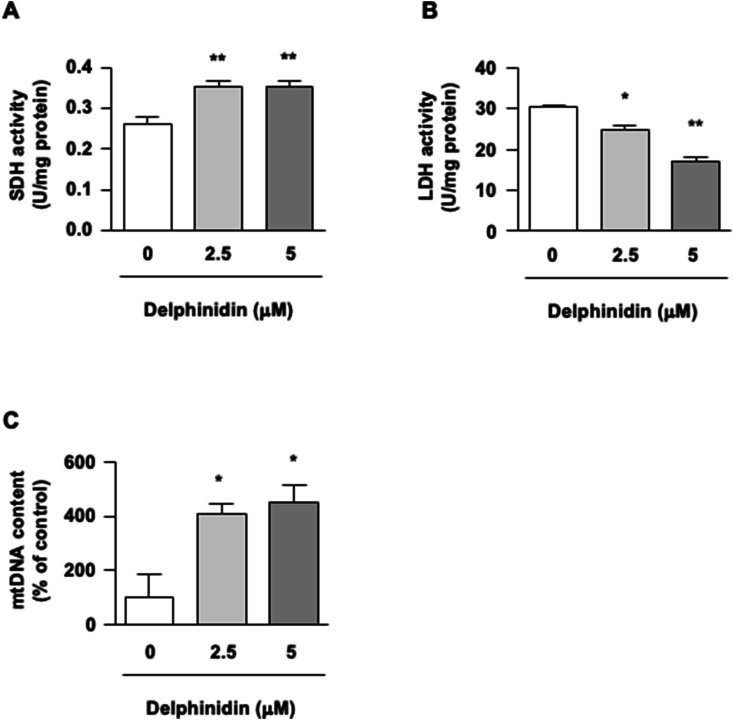
Effect of delphinidin on metabolic enzyme activities and mtDNA content in C2C12 myotubes. (A, B) C2C12 myotubes were incubated with delphinidin for 72 h, and the enzyme activities were measured using commercial kits. (C) Mitochondrial DNA (mtDNA) in C2C12 cells treated with delphinidin for 72 h was analyzed by qRT-PCR. Data are presented as mean ± SEM, *n* = 3. ∗*P* < 0.05 and ∗∗*P* < 0.01 as compared with 0 μM delphinidin.

Given that metabolic enzyme activities correlate with muscle fiber type, we examined the activities of SDH and LDH, which were markers of oxidative and glycolytic metabolism in skeletal muscle. In C2C12 myotubes, delphinidin induced SDH activation and suppressed LDH activity (Fig. 2A and B). Mitochondria are abundant in slow-twitch fibers but less in fast-twitch fibers [22]. To study the effect of delphinidin on the properties of muscle fiber types, we conducted qRT-PCR to measure mtDNA and found that delphinidin treatment increased the mtDNA content in C2C12 myotubes (Fig. 2C). Therefore, delphinidin induced a fast-to-slow muscle fiber type shift in C2C12 myotubes.

### 3.3 Delphinidin promotes muscle fiber type shift through AMPK in C2C12 myotubes

**Fig. 3:**
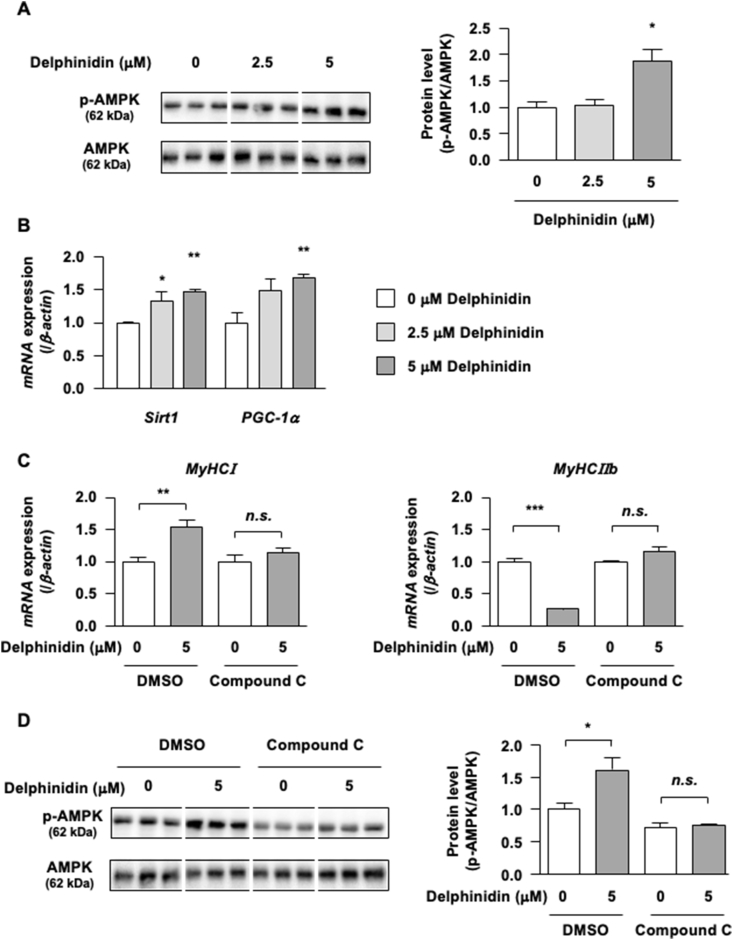
Delphinidin promotes muscle fiber type shift through AMPK in C2C12 myotubes. (A) C2C12 myotubes were treated with delphinidin for 1 h; subsequently, the phosphorylation of AMPK was assessed by western blotting and then normalized to AMPK. (B) After the C2C12 myotubes were treated with delphinidin for 48 h, Sirt1 and PGC-1α mRNA expression levels were measured by qRT-PCR. (C) C2C12 myotubes were treated with 5 μM delphinidin and 10 μM compound C separately or in combination for 48 h. Next, the mRNA expression levels of MyHCI and MyHCIIb were measured by qRT-PCR. (D) C2C12 myotubes were treated with 5 μM delphinidin and 10 μM compound C separately or in combination for 1 h. The protein level of *p*-AMPK was determined by western blotting analysis and normalized to AMPK. Data are presented as mean ± SEM, *n* = 3. ∗*P* < 0.05, ∗∗*P* < 0.01, and ∗∗∗*P* < 0.001 as compared with 0 μM delphinidin.

The activation of the AMPK pathway promotes mitochondrial biogenesis and skeletal muscle fiber type conversion. To elucidate the mechanism of muscle fiber type conversion induced by delphinidin, we examined AMPK involvement. Delphinidin significantly increased AMPK phosphorylation in C2C12 myotubes (Fig. 3A). In qRT-PCR analysis, delphinidin significantly upregulated the mRNA expression of Sirt1 and PGC-1α (Fig. 3B), which are downstream factors of AMPK signaling. To examine the involvement of AMPK in the regulation of muscle fiber type conversion by delphinidin, C2C12 myotubes were co-treated with delphinidin and compound C (AMPK inhibitor). Compound C effectively attenuated the increase in MyHCI mRNA expression and the suppression of delphinidin-induced MyHCIIb mRNA expression (Fig. 3C). Delphinidin upregulated *p*-AMPK protein level, whereas compound C attenuated the effect of delphinidin (Fig. 3D). Thus, delphinidin could regulate muscle fiber type shift through AMPK in C2C12 myotubes.

### 3.4 Effect of delphinidin on the upstream factors of AMPK signaling

**Fig. 4:**
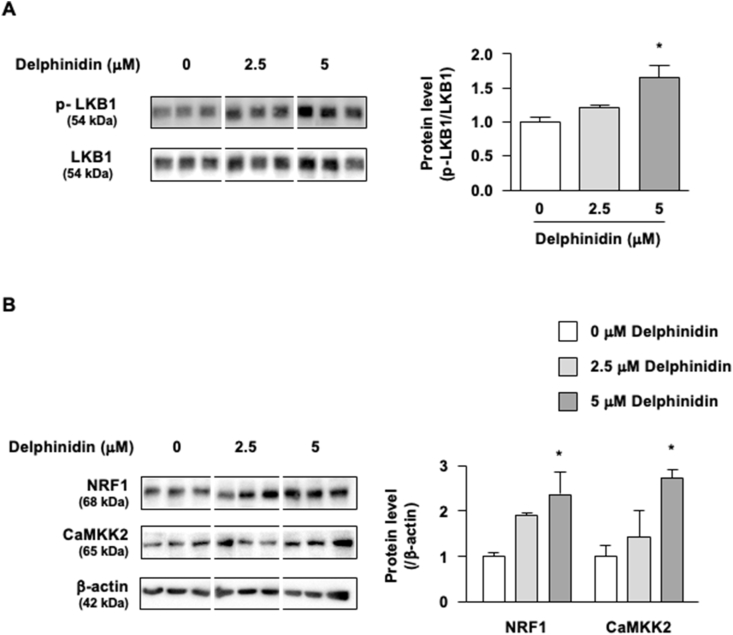
Delphinidin regulates the upstream the factors of AMPK signaling in C2C12 myotubes. (A, B) After the C2C12 myotubes were treated with delphinidin for 1 h, the protein level was assessed by western blotting. Data are presented as mean ± SEM, *n* = 3. ∗*P* < 0.05 as compared with 0 μM delphinidin.

AMPK is activated by phosphorylation, either by liver kinase B1 (LKB1) [23] or by an alternate pathway involving Ca^2+^-calmodulin-dependent kinase 2 (CaMKK2) [24]. Nuclear respiratory factor 1 (NRF1) binds to the CaMKK2 promoter and promote transcription, causing AMPK activation [25]. Our data showed that delphinidin increased LKB1 phosphorylation and NRF1 and CaMKK2 protein levels (Fig. 4A and B). Therefore, delphinidin could affect the upstream factors of AMPK activation.

## 4 Discussion

This study sought to elucidate the effect of delphinidin on skeletal muscle fiber type conversion. Results revealed that delphinidin induced muscle fiber type shift from fast-twitch to slow-twitch in C2C12 myotubes by activating the AMPK signaling pathway.

The higher the proportion of slow muscle fibers, the lower the risk of muscle-related diseases. The percentage of slow muscle fibers positively correlates with the sensitivity of insulin [26]. Some skeletal muscle change from slow-twitch to fast-twitch fibers in type 2 diabetes, decreasing the proportion of slow muscle fibers [27]. Furthermore, the rate of slow-twitch muscle fibers negatively correlates with muscle atrophy [28]. Therefore, by increasing the rate of slow muscle fibers, delphinidin may maintain muscle function and prevent muscle-related diseases. In addition, a high content of slow muscle fibers positively correlates with a high-quality meat [29]. Our results also indicated that delphinidin can potentially convert the meat quality of livestock. Although this study shows results using C2C12 myotubes, the effects of delphinidin using other muscle cells (rat L6 myotubes and human myoblast cell lines) need to be investigated. Furthermore, studies using an *in vivo* model is needed to elucidate the effect of delphinidin on muscle fiber type shift. To investigate the effects of delphinidin *in vivo*, we should perform an endurance test using a treadmill to assess running time and distance in mice.

AMPK is reportedly an important factor in regulating muscle fiber types conversion [30]. It has been demonstrated that AMPK promotes mitochondrial biogenesis by phosphorylating PGC-1α [31]. There is evidence that PGC-1α promote the expression of slow muscle fiber genes and results in oxidative fiber formation [19]. Our study suggested that delphinidin changes myofiber type and metabolic enzyme (SDH, LDH) activity by activating AMPK in C2C12 myotubes. AMPK activation regulates multiple physiological and pathological processes, and is involved in several diseases, including cancer [32], obesity [33], diabetes [34], and Alzheimer's disease [35]. Chen et al. reported that the AMPK signaling pathway is also involved in autophagy induced by delphinidin in breast cancer cells [36]. Delphinidin effectively reduced pathological myocardial hypertrophy via oxidative stress inhibition by activating AMPK [37]. Therefore, the AMPK signaling pathway may be a key factor responsible for the biological activity of delphinidin.

We identified delphinidin's effects on the upstream factors of AMPK. Delphinidin promoted LKB1 phosphorylation and increased CaMKK2 and NRF1 protein levels. LKB1 is activated by STE20-related adaptor protein and mouse protein 25 [38]. According to the study of Koh et al., PPARβ is a transcription factor for NRF1 and NRF1 increases CaMKK2 transcription and AMPK activation in the skeletal muscle [25]. Moreover, CaMKK2 is an enzyme activated by Ca^2+^/calmodulin binding [39], and delphinidin increases cytosolic calcium levels [40]. Our previous research found that NFATc3, activated by Ca^2+^, is involved in anti-atrophy effect of delphinidin [7]. Although the pathway for AMPK activation has already been widely studied, Ca^2+^ is likely to be a key factor as one of the molecules by which delphinidin activates AMPK.

Among the three anthocyanidins examined for their effects on muscle fiber types, only delphinidin increased the expression of MyHCI (slow-twitch) mRNA. Compared with cyanidin and pelargonidin, delphinidin has three hydroxyl groups on its B ring. Hydroxyl group increase has been associated with increased antioxidant capacity [41]. Delphinidin, cyanidin, and pelargonidin suppressed H_2_O_2_-induced lipid peroxidation [42] and LPS-induced COX-2 expression [43] in the order of delphinidin > cyanidin > pelargonidin. However, comparisons with other polyphenols and nutritional compounds are required to establish the muscle fiber type conversion effect of delphinidin. In addition, certain internal molecules mediate the biological effects of food compounds such as vitamins and polyphenols. Most vitamin A functions are mediated through two transcription factors, retinoic acid receptors (RARs) and retinoid X receptors (RXRs) [44]. Epigallocatechin-3-*O*-gallate (EGCG), one of the green tea catechin, has affinity with the cell surface protein 67 kDa laminin receptor [45], which mediates several physiological effects of EGCG [46]. Soy isoflavones show structural similarity to estrogen and exert estrogenic or anti-estrogenic effects through estrogen receptors [47]. The difference in the effects of anthocyanidins may be explained by the presence of a delphinidin-sensing molecule responsible for the function of delphinidin. Further research is required to elucidate the molecular mechanisms underlying delphinidin signaling.

In conclusion, delphinidin activated the AMPK signaling pathway, leading to muscle fiber type transformation. Thus, delphinidin has the potential to improve muscle function and prevent muscle-related diseases.

## CRediT authorship contribution statement

**Motoki Murata:** Writing – original draft, Validation, Investigation, Funding acquisition, Formal analysis, Data curation, Conceptualization. **Rina Takahashi:** Investigation, Formal analysis, Data curation. **Yuki Marugame:** Investigation, Formal analysis, Data curation. **Yoshinori Fujimura:** Writing – review & editing, Validation. **Hirofumi Tachibana:** Writing – review & editing, Supervision, Funding acquisition, Conceptualization.

## Data availability

Data will be made available on request.

## Funding sources

This work was supported by JSPS KAKENHI Grant Numbers JP22K14843 (to M.M.) and JP20H05683 (to H.T.).

## Declaration of competing interest

The authors declare that they have no known competing financial interests or personal relationships that could have appeared to influence the work reported in this paper.
