# Supplementary material for: Delphinidin induces a fast-to-slow muscle fiber type shift through the AMPK signaling pathway in C2C12 myotubes
Source: Biochem Biophys Rep. 2024 Nov 23;40:101884. doi: 10.1016/j.bbrep.2024.101884 (PMC11626064; doi:10.1016/j.bbrep.2024.101884)
Supplement: Multimedia component 1 [file mmc1.pptx]

## Slide 1
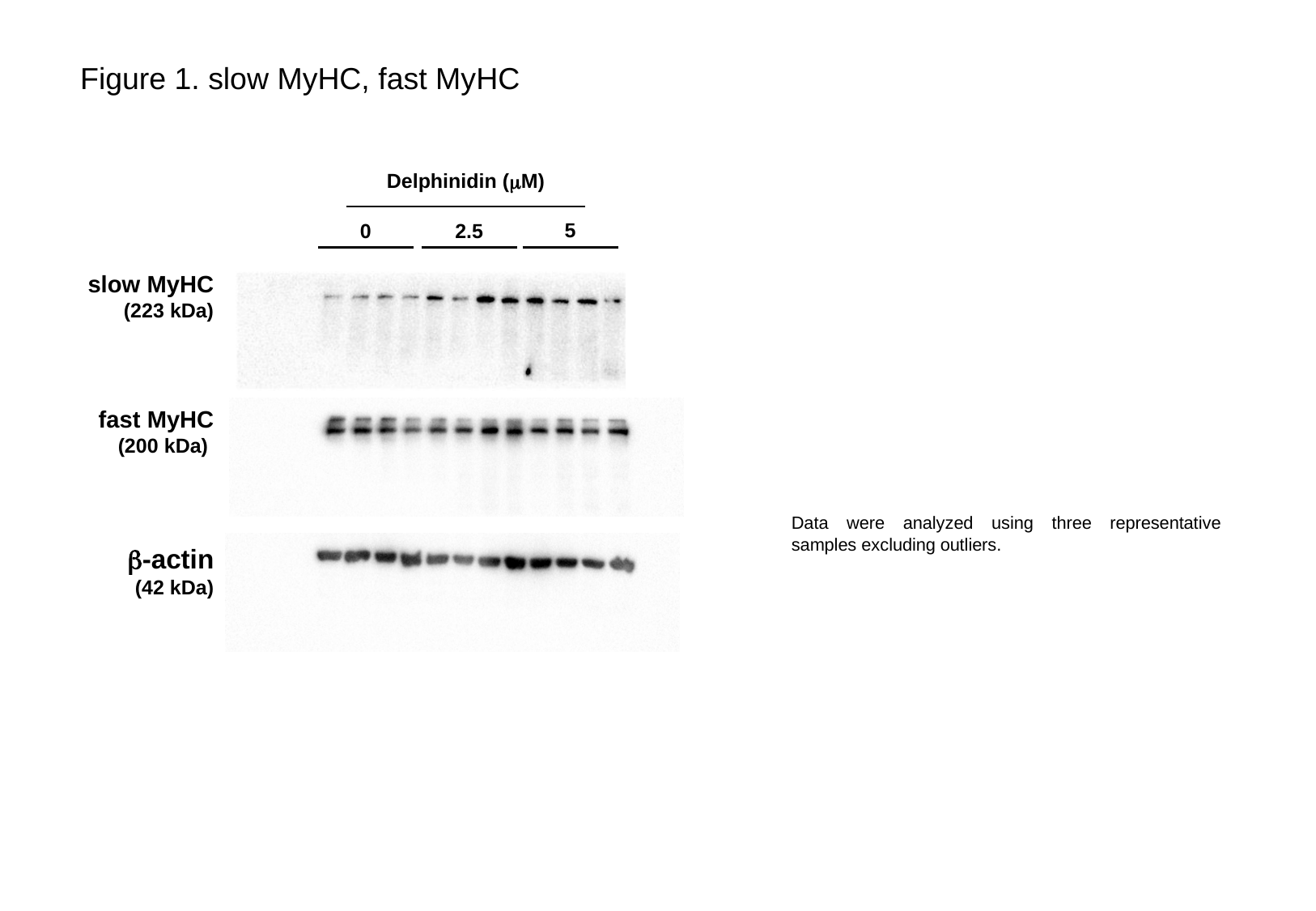

Figure 1. slow MyHC, fast MyHC
Delphinidin (mM)
5
0
2.5
slow MyHC
(223 kDa)
fast MyHC
(200 kDa)
Data were analyzed using three representative samples excluding outliers.
b-actin
(42 kDa)

## Slide 2
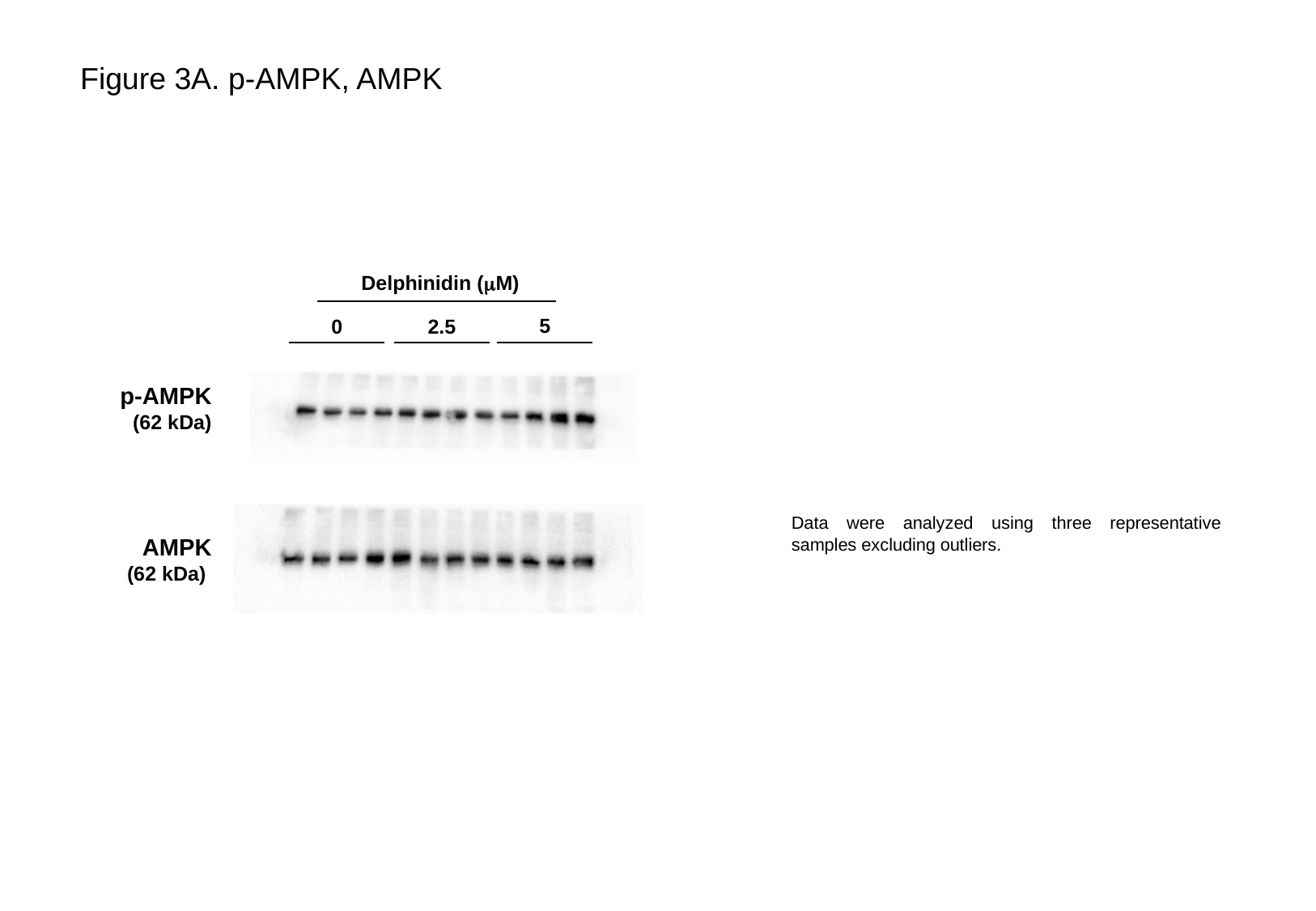

Figure 3A. p-AMPK, AMPK
Delphinidin (mM)
5
0
2.5
p-AMPK
(62 kDa)
Data were analyzed using three representative samples excluding outliers.
AMPK
(62 kDa)

## Slide 3
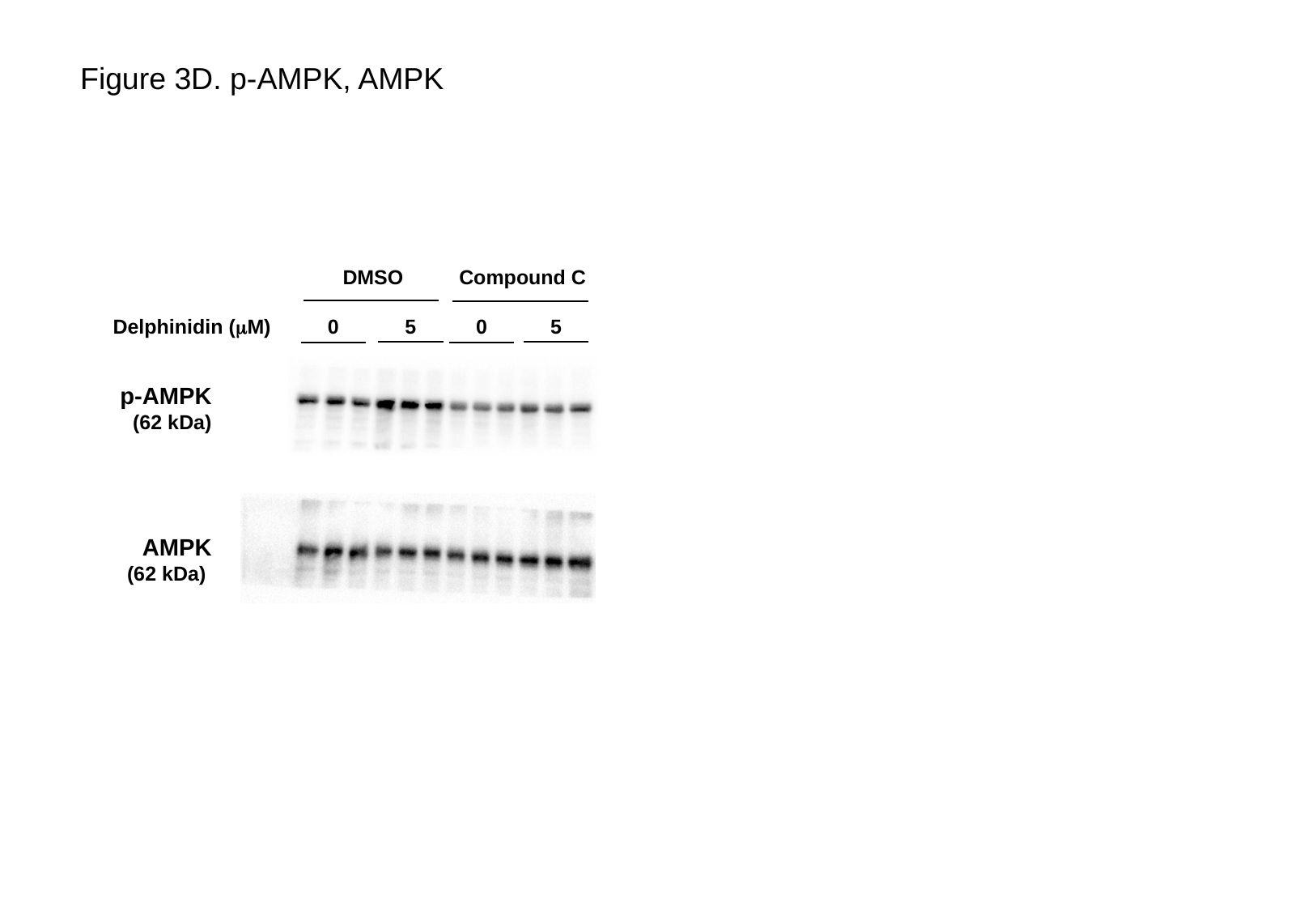

Figure 3D. p-AMPK, AMPK
Compound C
DMSO
Delphinidin (mM)
0
5
0
5
p-AMPK
(62 kDa)
AMPK
(62 kDa)

## Slide 4
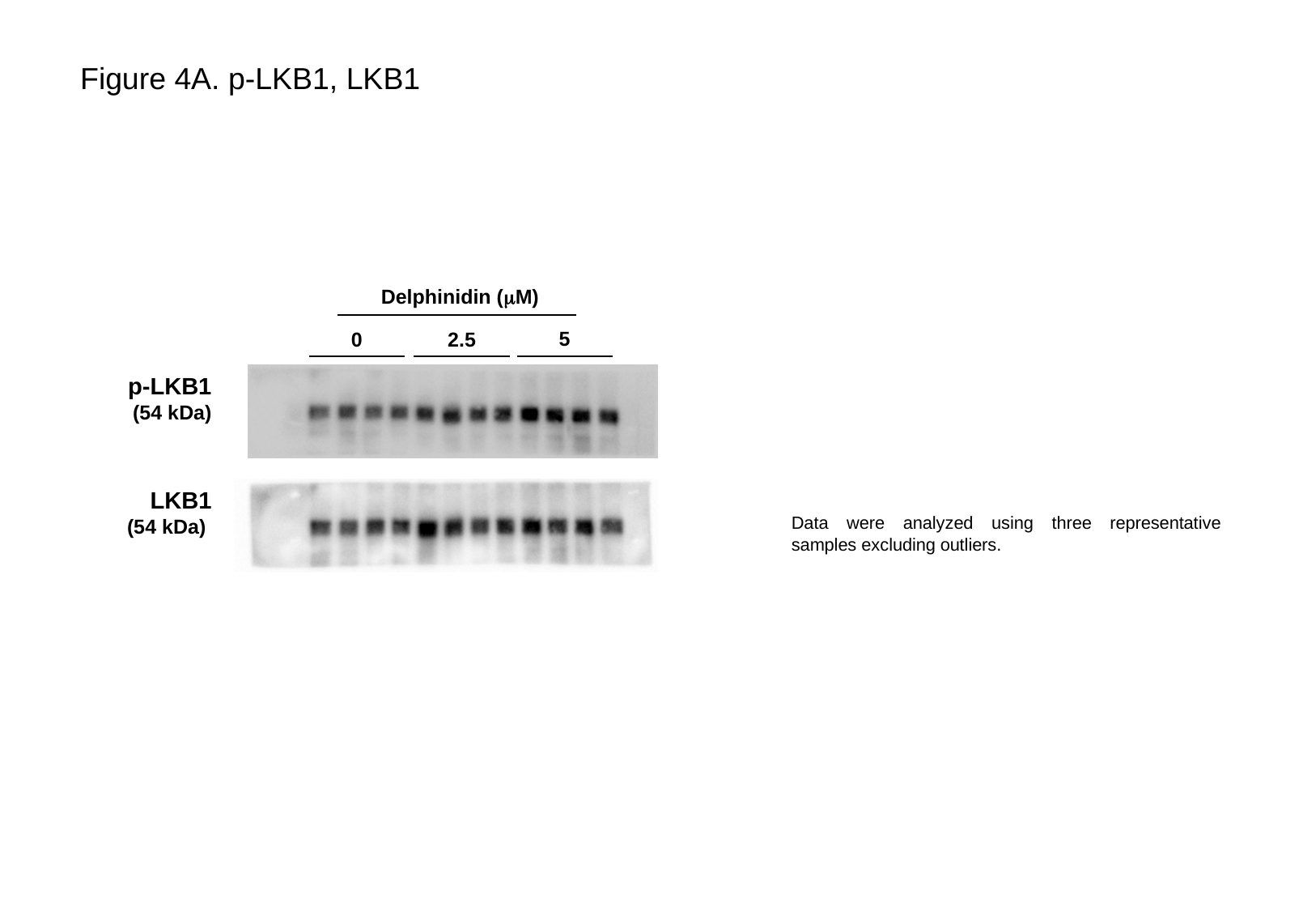

Figure 4A. p-LKB1, LKB1
Delphinidin (mM)
5
0
2.5
p-LKB1
(54 kDa)
LKB1
(54 kDa)
Data were analyzed using three representative samples excluding outliers.

## Slide 5
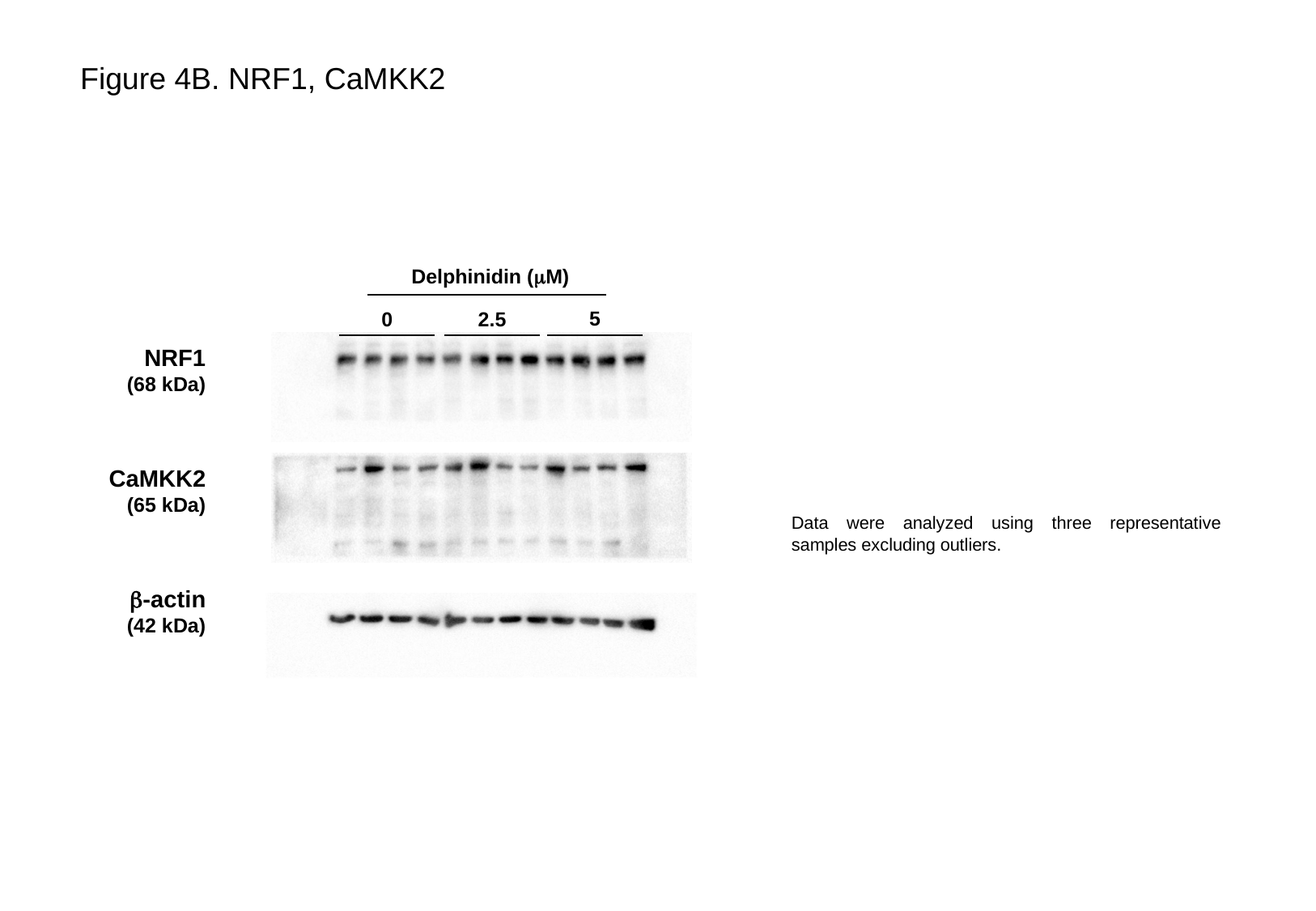

Figure 4B. NRF1, CaMKK2
Delphinidin (mM)
5
0
2.5
NRF1
(68 kDa)
CaMKK2
(65 kDa)
Data were analyzed using three representative samples excluding outliers.
b-actin
(42 kDa)
