# Supplementary material for: Delphinidin induces a fast-to-slow muscle fiber type shift through the AMPK signaling pathway in C2C12 myotubes
Source: Biochem Biophys Rep. 2024 Nov 23;40:101884. doi: 10.1016/j.bbrep.2024.101884 (PMC11626064; doi:10.1016/j.bbrep.2024.101884)
Supplement: Multimedia component 2 [file mmc2.docx]

**Table S1 Sequences of specific primers**

| Speasies | Gene | Sequense (5'-3') |
| --- | --- | --- |
| Mouse | MyHCI-forward | CTTCTACAGGCCTGGGCTTAC |
| Mouse | MyHCI-reverse | CTCCTTCTCAGACTTCCGCAG |
| Mouse | MyHCIIa-forward | TTCCAGAAGCCTAAGGTGGTC |
| Mouse | MyHCIIa-reverse | GCCAGCCAGTGATGTTGTAAT |
| Mouse | MyHCIIx-forward | CAACCCATACGACTACGCCT |
| Mouse | MyHCIIx-reverse | CATCAGAAGTGAAGCCCAGAAT |
| Mouse | MyHCIIb-forward | CTTGTCTGACTCAAGCCTGCC |
| Mouse | MyHCIIb-reverse | TCGCTCCTTTTCAGACTTCCG |
| Mouse | TNNC1-forward | AACACAAAGTCGCAGATGAGGATTC |
| Mouse | TNNC1-reverse | GCTGCTCTCACGCCATTCTGTA |
| Mouse | TNNI1-forward | CCAATTATGAGATGGACTGTGGGTA |
| Mouse | TNNI1-reverse | CTTGGGAATGGTGCAGTGTAATCTA |
| Mouse | SIRT1-forward | ACAGTGACAGTGGCACATGC |
| Mouse | SIRT1-reverse | AATCCAGATCCTCCAGCACA |
| Mouse | PGC-1α-forward | GGATTCAGACTACAATATAGC |
| Mouse | PGC-1α-reverse | CATGTATGAGACCAAGCGTC |
| Mouse | β-actin-forward | CATCCGTAAAGACCTCTATGCCAAC |
| Mouse | β-actin-reverse | ATGGAGCCACCGATCCACA |
